# Denosumab Versus Zoledronic Acid for Muscle Strength and Physical Performance in Postmenopausal Osteoporosis: A 3‐Year Prospective Study

**DOI:** 10.1002/jcsm.70406

**Published:** 2026-10-21

**Authors:** Xiangwen Li, Zineng Yuan, Nina Ma, Bijuan Huang, Hongyue Tao, Shuang Chen

**Affiliations:** ^1^ Department of Radiology Huashan Hospital, Fudan University Shanghai China; ^2^ Clinical Medical College Fudan University Shanghai China; ^3^ Department of Clinical Nutrition Huashan Hospital, Fudan University Shanghai China; ^4^ Department of Radiology Fuzhou Second Hospital Fuzhou China

**Keywords:** computed tomography, OPG/sRANKL, osteoporosis, sarcopenia

## Abstract

**Background:**

Osteoporosis and sarcopenia frequently coexist in older adults, yet whether denosumab provides muscle‐related benefits beyond bone remains uncertain. We compared denosumab and zoledronic acid across functional, imaging, biologic and study‐defined sarcopenia outcomes based on an Asian Working Group for Sarcopenia (AWGS) 2025‐informed framework and examined whether early osteoprotegerin (OPG)/soluble receptor activator of nuclear factor‐kappa B ligand (sRANKL) responses tracked with later benefit.

**Methods:**

This prospective observational cohort study included 186 postmenopausal women with osteoporosis and prescreened sarcopenia risk (82 denosumab, 104 zoledronic acid). Assessments at baseline and 12, 24 and 36 months included grip strength, gait speed, 5‐times chair stand, lumbar spine and femoral neck bone mineral density (BMD), dual‐energy X‐ray absorptiometry (DXA)‐derived appendicular skeletal muscle mass index (ASMI), computed tomography (CT)‐derived T12‐L4 skeletal muscle volume index (SMVI), skeletal muscle density (SMD), intermuscular fat volume index (IMFI), visceral fat volume index (VFI) and OPG/sRANKL‐centred biologic markers. Continuous outcomes were analysed using linear mixed‐effects models, categorical outcomes with Fisher exact tests and responder and mediation analyses.

**Results:**

Denosumab showed greater functional improvement than zoledronic acid. At 12 months, grip strength increased by 1.45 ± 0.51 versus 0.66 ± 0.34 kg, gait speed by 0.041 ± 0.013 versus 0.017 ± 0.011 m/s and 5‐times chair stand time by −0.82 ± 0.27 versus −0.32 ± 0.18 s (all *p* < 0.001). At 36 months, changes were 2.52 ± 0.63 versus 0.99 ± 0.45 kg, 0.070 ± 0.017 versus 0.027 ± 0.012 m/s and −1.47 s (−1.63, −1.34) versus −0.63 s (−0.82, −0.50) (all *p* < 0.001). ASMI did not differ at 12 months (*p* = 0.119) but favoured denosumab at 24 and 36 months (both *p* < 0.001; 36‐month change, 0.192 ± 0.059 vs. 0.089 ± 0.050 kg/m^2^). Sarcopenia prevalence was lower with denosumab at 12, 24 and 36 months (52.1% vs. 69.8%, 30.2% vs. 50.7% and 20.0% vs. 46.0%), and remission was higher (36.7% vs. 15.5%, 63.5% vs. 37.3% and 75.0% vs. 37.0%). Denosumab also produced greater increases in SMVI and SMD and greater reductions in IMFI and VFI. The OPG/sRANKL ratio increased more strongly at 12 months (2.503 [2.053, 3.429] vs. 0.807 [0.549, 0.994], *p* < 0.001). High responders had larger 36‐month gains in grip strength (2.80 ± 0.65 vs. 2.21 ± 0.44 kg, *p* < 0.001) and CT‐defined SMVI (2.816 ± 0.861 vs. 2.199 ± 0.524, *p* = 0.002). Twelve‐month OPG/sRANKL change showed a pattern statistically consistent with partial mediation of the association between treatment group and 36‐month grip strength (37.0%) and CT‐defined SMVI (39.9%).

**Conclusions:**

Compared with zoledronic acid, denosumab was associated with stronger early OPG/sRANKL activation, earlier functional improvement, more favourable CT‐defined remodelling, later DXA‐derived muscle‐mass gain and lower study‐defined sarcopenia burden based on an AWGS 2025‐informed framework over 3 years. These findings support validation of RANKL inhibition in osteosarcopenic phenotypes.

## 1 Introduction

Osteoporosis and sarcopenia commonly coexist in older adults and, when present together as osteosarcopenia, are associated with greater risks of falls, fractures, disability, and loss of independence than either disorder alone [1, 2, 3]. Recent Asian consensus documents have also shifted the clinical focus from muscle mass alone toward strength, physical performance and broader muscle health across the life course [4, 5, 6, 7]. This reframing is particularly relevant in postmenopausal osteoporosis, where improving bone mineral density (BMD) alone may not fully address the functional vulnerability that drives frailty and future fracture risk [8].

Denosumab is an established antiresorptive therapy for postmenopausal osteoporosis, with robust evidence for suppressing bone turnover, increasing BMD and reducing fracture risk [9, 10, 11]. Comparative meta‐analytic evidence also supports its efficacy against commonly used bisphosphonate regimens [12]. However, the biologic effects of receptor activator of nuclear factor‐kappa B ligand (RANKL) inhibition may extend beyond bone. Recent reviews of bone–muscle crosstalk have highlighted the receptor activator of nuclear factor‐kappa B (RANK)/RANKL/osteoprotegerin (OPG) axis as a plausible signalling node linking skeletal remodelling, muscle metabolism, inflammation and endocrine adaptation [13, 14, 15]. Experimental work further shows that skeletal muscle expresses components of this pathway and that RANK signalling influences calcium handling, sarcoplasmic/endoplasmic reticulum Ca2+‐ATPase activity, oxidative programming and muscle performance [16, 17].

Preclinical and translational studies have also shown that RANKL inhibition can improve muscle strength and insulin sensitivity, supporting the concept that denosumab might have clinically relevant musculoskeletal effects beyond fracture prevention [18, 19]. Nevertheless, current human evidence remains heterogeneous, and recent systematic synthesis indicates that the signal for benefit is more consistent for strength and physical performance than for lean‐mass endpoints [20]. Most prior studies have evaluated isolated outcomes rather than integrating biologic response, physical performance, dual‐energy X‐ray absorptiometry (DXA)‐derived muscle mass, computed tomography (CT)‐based body composition and formal Asian Working Group for Sarcopenia (AWGS)‐based sarcopenia end points within the same longitudinal framework. We therefore compared denosumab with zoledronic acid across bone, muscle, metabolic and imaging domains and further tested whether early OPG/soluble receptor activator of nuclear factor‐kappa B ligand (sRANKL) responses tracked with later structural and functional benefit.

As a result, it remains unclear whether changes in the OPG/sRANKL axis translate into clinically meaningful structural and functional muscle benefit in routine practice. In this longitudinal study, we compared denosumab and zoledronic acid with respect to muscle strength, physical performance, DXA‐ and CT‐based body composition and sarcopenia outcomes based on an AWGS 2025‐informed framework and the prespecified study definition. We also examined whether these changes were accompanied by shifts in the OPG/sRANKL axis and related metabolic markers and whether patients with a greater biologic response experienced greater clinical benefit. We hypothesized that compared with zoledronic acid, denosumab would lead to more favourable adaptations across bone, muscle and metabolic domains and that patients with larger OPG/sRANKL responses would show the greatest structural and functional improvement.

## 2 Materials and Methods

This prospective observational cohort study was approved by the Health Sciences Institutional Review Board of Huashan Hospital, Fudan University (HIRB; approval no. 2019‐410) and was conducted in accordance with the 1964 Declaration of Helsinki and its later amendments. All participants provided written informed consent before enrollment. Because this was an observational cohort in which treatment was determined as part of routine clinical care rather than assigned by the study protocol, the study was not registered as a clinical trial.

### 2.1 Participants

Between March 2020 and April 2022, we prospectively screened 311 consecutive postmenopausal women aged 50 years or older with confirmed osteoporosis who initiated denosumab or zoledronic acid. At study initiation, participant screening and data collection were conducted within the contemporaneous AWGS 2019 framework [21]. Screening was performed in women with prespecified risk factors for sarcopenia, most commonly low handgrip strength at screening. All eligible participants underwent a complete baseline assessment, including BMD, whole‐body DXA, muscle strength, physical performance, laboratory testing and CT‐based body composition analysis.

Participants were excluded if written informed consent could not be obtained (*n* = 33); if they had secondary osteoporosis requiring disease‐specific treatment (*n* = 8); active malignancy receiving systemic therapy (*n* = 10); inflammatory myopathy or another neuromuscular disease (*n* = 7); severe hepatic dysfunction (*n* = 4); renal insufficiency precluding standard antiresorptive therapy (*n* = 11); long‐term systemic glucocorticoid exposure (> 5 mg/day prednisone equivalent for > 3 months) (*n* = 6); use of anabolic bone agents within 12 months (*n* = 6); prior denosumab or intravenous bisphosphonate exposure within 12 months before enrollment (*n* = 5); a recent fragility fracture, major surgery or hospitalization within 3 months before baseline (*n* = 7); inability to complete functional testing (*n* = 7); or missing baseline OPG or sRANKL measurements (*n* = 21). After these exclusions, 186 participants were included in the final analysis.

### 2.2 Treatment Exposure

Treatment assignment was made by the treating physician as part of routine clinical care rather than by the study protocol. Treatment selection considered the individual fracture‐risk profile, renal function, previous osteoporosis‐treatment history, including intolerance or inadequate response to bisphosphonate therapy, patient preference regarding route and frequency of administration and the anticipated ability to maintain scheduled treatment. Anticipated treatment continuity was defined as the documented ability at baseline to adhere to scheduled dosing and follow‐up visits throughout the planned treatment period. Treating physicians had access to routine clinical information required for osteoporosis management, whereas research‐derived CT body‐composition measurements, AWGS‐based sarcopenia classifications and exploratory muscle‐related biomarker results were not used for treatment selection. Patients in the denosumab group received 60 mg subcutaneously every 6 months, whereas those in the zoledronic acid group received 5 mg intravenously once yearly over at least 15 min. Unless contraindicated, all participants were advised to take calcium and vitamin D supplements, and supplement use was recorded prospectively at each study visit. These treatment schedules reflected standard prescribing practice during the study period.

### 2.3 Clinical, Functional and Event Assessments

At baseline and at each follow‐up visit, we collected demographic and clinical data, including age, height, weight, body mass index, history of prior fragility fracture, calcium supplementation, vitamin D supplementation, interval falls and new fragility fractures. Falls were defined as any unintentional event resulting in coming to rest on the ground or a lower level since the previous visit, and new fragility fractures were confirmed radiographically. For descriptive event analyses, cumulative frequencies at 12, 24 and 36 months were defined as the proportion of participants who had experienced at least 1 corresponding event from baseline to the respective visit.

Handgrip strength was assessed in the dominant hand with a calibrated handheld dynamometer (T.K.K.5401 GRIP‐D; Takei Scientific Instruments Co. Ltd., Tokyo, Japan). The highest value from three trials was recorded. Usual gait speed was assessed over a 10‐m walkway at baseline and at each follow‐up visit, and the better of 2 trials was recorded. At the same time points, participants also completed the 5‐times chair stand test using a standard‐height chair without armrests; the total time required to complete five rises with the arms folded across the chest was recorded.

Regular physical activity, resistance exercise and physical therapy or rehabilitation were recorded at baseline and at each follow‐up visit. Regular physical activity reflected participation in routine moderate‐intensity ambulatory or leisure‐time physical activity, whereas resistance exercise referred to structured muscle‐strengthening exercise. Physical therapy or rehabilitation was recorded when participants received supervised rehabilitation or physical therapy during the corresponding follow‐up interval. General recommendations regarding resistance exercise were provided to participants in both treatment groups, but no protocol‐mandated or supervised resistance‐training program was implemented. When clinically indicated, physical therapy or rehabilitation was provided at designated rehabilitation institutions and was recorded during follow‐up.

### 2.4 DXA Assessment and Sarcopenia Classification

DXA was performed using a Lunar Prodigy Pro Compact system (GE Healthcare). Lumbar spine (L1‐L4) and femoral neck BMD values were recorded in g/cm^2^. Whole‐body DXA was used to derive appendicular lean mass, and appendicular skeletal muscle mass index (ASMI) was calculated as appendicular lean mass divided by height squared (kg/m^2^). To reduce interscan variability, serial DXA examinations were performed on the same machine.

Sarcopenia‐related outcomes were re‐evaluated according to the updated AWGS 2025 framework during the final analysis [4]. Although participant enrollment and the original study assessments were conducted before publication of the AWGS 2025 consensus, all variables required for the updated classification, including age, handgrip strength and DXA‐derived ASMI, had been prospectively collected at baseline and at each scheduled follow‐up visit. Therefore, the updated AWGS 2025 framework could be applied retrospectively using the previously collected study data. Sarcopenia was defined by the concurrent presence of low muscle strength and low muscle mass according to the age‐specific AWGS 2025 criteria.

### 2.5 CT Acquisition and Body Composition Analysis

CT‐based body composition analysis was performed on clinically acquired whole‐abdomen CT examinations obtained within ±3 months of each scheduled study time point. Scans were acquired on multidetector CT systems (SOMATOM Definition Flash; Siemens Healthineers). For longitudinal analyses, each participant was scanned on the same CT system with the same acquisition protocol across time points.

Automated segmentation was performed with TotalSegmentator (version 2.13.0; University Hospital Basel), an open‐source deep learning tool for whole‐body CT segmentation [22]. A musculoskeletal radiologist with 10 years of experience, blinded to treatment group and laboratory results, reviewed all segmentation outputs, and manual correction was performed when necessary.

Body composition was assessed using a volumetric T12‐L4 analysis. Prospectively extracted CT‐derived metrics included T12‐L4 skeletal muscle volume index (SMVI), subcutaneous fat volume index (SFI), visceral fat volume index (VFI), intermuscular fat volume index (IMFI) and skeletal muscle density (SMD). All volume‐based indices were normalized to height squared.

### 2.6 Laboratory Assessment

Fasting venous blood samples were collected in the morning after an overnight fast at baseline and at each follow‐up visit. Serum soluble receptor activator of nuclear factor‐κB ligand (sRANKL) and OPG were measured with enzyme‐linked immunosorbent assay kits (R&D Systems; Catalogue #DY805 and #DY626), and the OPG/sRANKL ratio was used as the main marker of biologic response. Bone turnover markers included serum C‐terminal telopeptide of type I collagen (CTX) and procollagen type I N‐terminal propeptide (P1NP). For baseline categorical summaries, high sRANKL was prespecified as a baseline value at or above the cohort median.

The laboratory panel also included prospectively collected muscle‐ and metabolism‐related biomarkers: myostatin, follistatin, the follistatin‐to‐myostatin ratio, brain‐derived neurotrophic factor (BDNF), procollagen type III N‐terminal peptide (P3NP), insulin‐like growth factor 1 (IGF‐1), irisin, high‐sensitivity C‐reactive protein (hs‐CRP), interleukin‐6 (IL‐6), tumour necrosis factor alpha (TNF‐α), fasting glucose, fasting insulin, the homeostasis model assessment of insulin resistance (HOMA‐IR), creatinine, cystatin C and the sarcopenia index (creatinine/cystatin C × 100).

### 2.7 Study Outcomes

The primary outcomes were longitudinal changes in muscle strength and physical performance, including grip strength, gait speed and 5‐times chair stand time. Secondary outcomes included longitudinal changes in lumbar spine and femoral neck BMD, whole‐body DXA‐derived ASMI, CT‐derived body composition metrics (SMVI, SFI, VFI, IMFI and SMD), biologic markers, low muscle strength, low muscle mass and sarcopenia status re‐evaluated according to the AWGS 2025 framework, as well as interval falls and new fragility fractures. Categorical follow‐up outcomes also included sarcopenia remission among participants with sarcopenia at baseline and baseline high‐sRANKL status. For the responder and mediation analyses, the representative functional and structural response end points were 36‐month grip‐strength gain and 36‐month CT‐defined SMVI gain.

Within the denosumab group, high biologic response was prespecified as a 12‐month increase in the OPG/sRANKL ratio at or above the within‐group median; the remaining participants were classified as low responders.

### 2.8 Statistical Analysis

Continuous variables were assessed for normality with the Shapiro–Wilk test and are reported as mean ± SD when approximately normally distributed in both groups and as median (Q1, Q3) otherwise. Categorical variables are reported as number (percentage). Baseline between‐group comparisons were performed with Welch *t*‐tests, Mann–Whitney *U* tests or Fisher exact tests, as appropriate.

For longitudinal analyses of continuous outcomes, change‐from‐baseline summaries were generated at 12, 24 and 36 months. Treatment effects were evaluated with linear mixed‐effects models for the major continuous outcomes, including functional measures, lumbar spine and femoral neck BMD, ASMI, CT‐derived body composition metrics and biologic markers. These models included fixed effects for treatment group, categorical follow‐up visit and the group‐by‐time interaction, along with a subject‐specific random intercept. Sensitivity analyses used adjusted linear mixed‐effects models that further included age, baseline vitamin D supplementation and baseline variables that differed between groups. In an additional sensitivity analysis of the primary functional outcomes, regular physical activity, resistance exercise and physical therapy or rehabilitation were included as time‐varying covariates in the linear mixed‐effects models to account for potential lifestyle‐related confounding.

To further address potential confounding related to treatment selection, a propensity score‐weighted sensitivity analysis was performed. The propensity score for receipt of denosumab was estimated using multivariable logistic regression including age, prior fragility fracture, baseline lumbar spine and femoral neck BMD, estimated creatinine clearance, baseline vitamin D supplementation, prior bisphosphonate intolerance or inadequate response, anticipated treatment continuity and administration preference. A stabilized inverse probability of treatment weighting (IPTW) approach was used, with weights derived from the propensity scores. Covariate balance before and after weighting was assessed using standardized mean differences (SMDs), with an absolute SMD < 0.10 considered indicative of adequate balance. IPTW‐weighted between‐group differences in changes in grip strength, gait speed and 5‐times chair stand time were then estimated at 12, 24 and 36 months with 95% confidence intervals.

For categorical longitudinal outcomes, including low muscle strength, low muscle mass, sarcopenia status, sarcopenia remission among participants with sarcopenia at baseline, interval falls and new fragility fractures, proportions were compared between groups at each follow‐up visit with Fisher exact tests. Cumulative event frequencies were summarized descriptively across follow‐up. Responder‐stratified categorical outcomes within the denosumab group were also compared with Fisher exact tests.

To examine whether improvement in muscle strength was associated with changes in BMD, correlations between change in grip strength and changes in lumbar spine and femoral neck BMD were evaluated at 12, 24 and 36 months. Analyses were performed in the pooled cohort and separately within each treatment group; additional linear regression models adjusted for treatment group and age.

To examine whether the treatment‐associated functional differences varied by age, an exploratory subgroup analysis was performed according to age at baseline (50–64 years vs. ≥ 65 years). Between‐group differences in 36‐month changes in grip strength, gait speed and 5‐times chair stand time were estimated within each age stratum, and treatment‐by‐age‐stratum interaction terms were tested.

Because the biologic hypothesis of the study was centered on the OPG/sRANKL axis, we assessed associations between changes in biologic markers and functional or imaging outcomes using Pearson correlation coefficients, primarily within the denosumab group, to determine whether early pathway activation was associated with later structural and functional benefit.

We also performed a prespecified responder analysis within the denosumab group. High biologic responders were defined as participants with a 12‐month increase in the OPG/sRANKL ratio at or above the within‐group median; the remaining participants were classified as low responders. Functional outcomes, DXA‐derived muscle mass, CT‐derived body composition variables, metabolic markers and categorical sarcopenia outcomes were then compared between responder strata over follow‐up.

Exploratory mediation analyses examined whether the 12‐month change in the OPG/sRANKL ratio mediated the association between treatment group and 36‐month muscle‐related outcomes, including grip‐strength gain and CT‐defined SMVI gain. Indirect, direct and total effects, along with the proportion mediated, were estimated by bootstrap resampling with 5000 iterations and 95% confidence intervals. No multiplicity adjustment was applied to secondary or exploratory analyses. All tests were two‐sided, and *p* < 0.05 was considered statistically significant. Statistical analyses were performed with R software (version 4.3.2) and Python (version 3.11.9).

## 3 Results

The overall study design, multimodal assessment framework and longitudinal evaluation schedule are shown in Figure 1. The final cohort included 186 postmenopausal women, with 82 assigned to denosumab and 104 to zoledronic acid. Baseline characteristics were generally similar across the main clinical, densitometric, functional and study‐defined sarcopenia variables under the AWGS 2025‐informed framework (Table 1). The high baseline prevalence of low muscle strength and sarcopenia was expected, as the cohort was intentionally enriched for women with prescreened sarcopenia risk rather than sampled from an unselected osteoporosis population.

**FIGURE 1:**
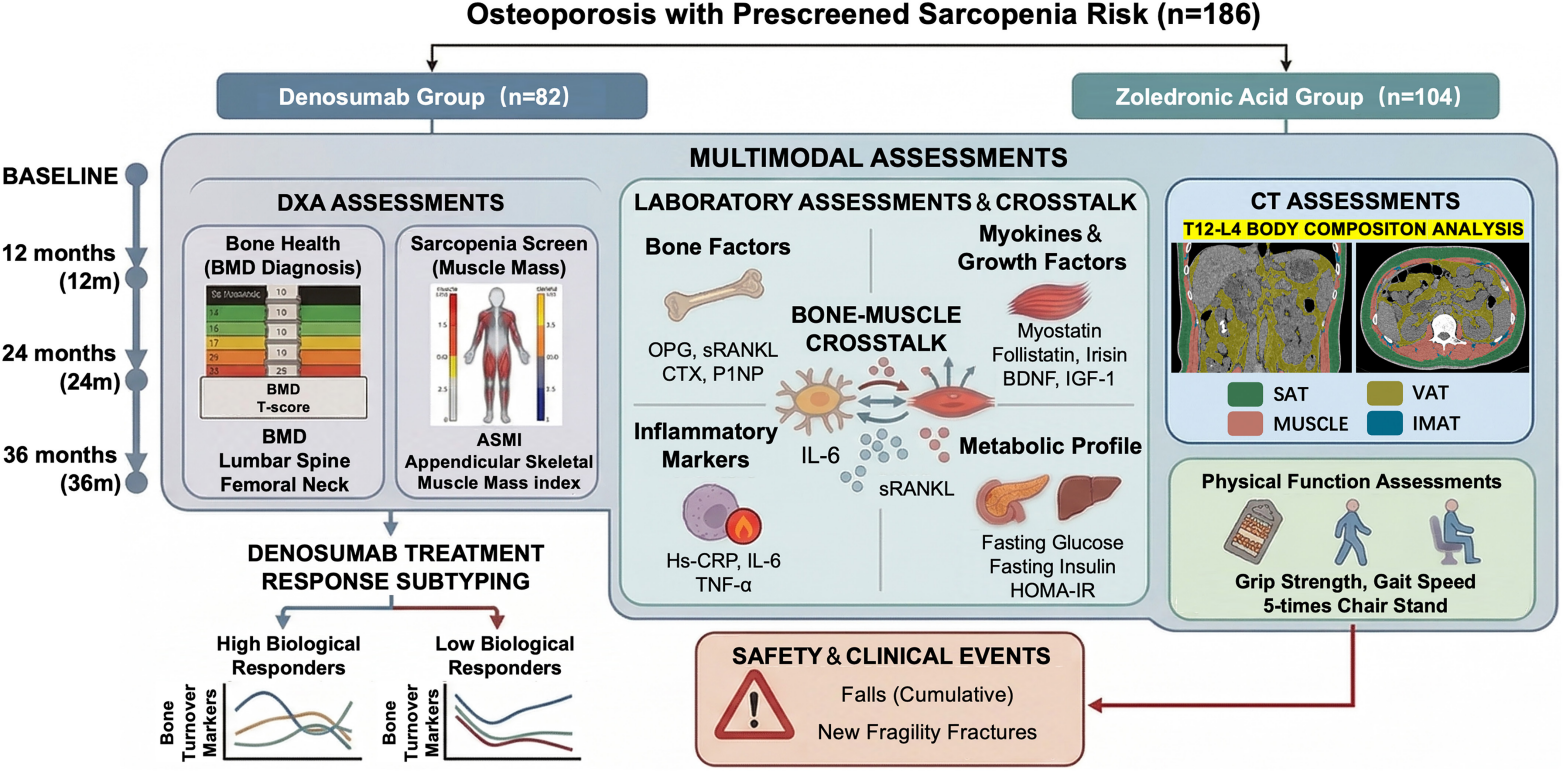
Study design and multimodal assessment framework.

**TABLE 1:** Baseline clinical, functional, biochemical and CT‐derived body composition characteristics of the study cohort.

Variable	Denosumab (*n* = 82)	Zoledronic acid (*n* = 104)	*p*
Demographics			
Age, years	64.6 ± 3.5	65.8 ± 5.5	0.076
Female sex, *n* (%)	82/82 (100.0%)	104/104 (100.0%)	—
Calcium supplementation, *n* (%)	82/82 (100.0%)	104/104 (100.0%)	1.000
Vitamin D supplementation, *n* (%)	80/82 (97.6%)	93/104 (89.4%)	0.041*
Height, m	1.558 (1.534, 1.604)	1.561 (1.540, 1.602)	0.711
Weight, kg	58.4 (52.2, 64.0)	58.6 (50.8, 68.0)	0.689
BMI, kg/m^2^	23.5 (21.4, 25.9)	23.4 (20.9, 27.2)	0.576
DXA			
Lumbar spine BMD, g/cm^2^	0.706 (0.681, 0.738)	0.692 (0.653, 0.748)	0.255
Femoral neck BMD, g/cm^2^	0.613 ± 0.036	0.617 ± 0.035	0.550
Whole‐body DXA ASMI, kg/m^2^	5.098 (4.987, 5.243)	5.210 (4.997, 5.322)	0.105
Function			
Grip strength, kg	15.92 (15.10, 17.10)	15.72 (14.71, 16.70)	0.266
Gait speed, m/s	0.883 (0.852, 0.910)	0.885 (0.847, 0.921)	0.772
5‐times chair stand, s	14.47 (13.84, 15.02)	14.43 (13.88, 15.23)	0.550
RANKL/OPG and bone turnover			
sRANKL, pmol/L	1.105 (0.977, 1.213)	1.086 (0.961, 1.217)	0.682
OPG, pmol/L	4.885 (4.621, 5.225)	4.902 (4.534, 5.219)	0.679
OPG/sRANKL ratio	4.487 (4.036, 5.048)	4.404 (4.016, 5.143)	0.966
CTX, ng/mL	0.712 (0.625, 0.770)	0.726 (0.639, 0.782)	0.696
P1NP, ng/mL	53.00 (48.82, 57.30)	56.70 (49.08, 62.90)	0.125
Myokines/laboratory			
Myostatin, ng/mL	4.045 ± 0.242	4.061 ± 0.221	0.643
Follistatin, ng/mL	1.550 ± 0.087	1.571 ± 0.089	0.106
Follistatin/myostatin ratio	0.386 (0.365, 0.409)	0.383 (0.366, 0.412)	0.989
Irisin, ng/mL	3.670 ± 0.204	3.698 ± 0.258	0.424
BDNF, ng/mL	19.89 ± 1.44	20.35 ± 1.58	0.040*
P3NP, μg/L	4.562 (4.502, 4.749)	4.687 (4.544, 4.836)	0.021*
IGF‐1, ng/mL	104.7 (99.1, 111.6)	105.2 (98.7, 112.2)	0.990
Inflammation			
hs‐CRP, mg/L	3.168 (2.779, 3.857)	3.388 (2.755, 3.865)	0.694
IL‐6, pg./mL	4.071 ± 0.800	4.042 ± 0.936	0.817
TNF‐α, pg./mL	2.608 ± 0.259	2.693 ± 0.308	0.044*
Metabolic			
Fasting glucose, mmol/L	5.420 ± 0.280	5.447 ± 0.326	0.553
Fasting insulin, uIU/mL	8.496 ± 2.005	8.818 ± 2.384	0.318
HOMA‐IR	2.032 (1.732, 2.428)	2.043 (1.579, 2.687)	0.388
Laboratory			
Creatinine, mg/dL	0.700 (0.633, 0.778)	0.688 (0.619, 0.799)	0.990
Cystatin C, mg/L	0.953 (0.837, 1.070)	1.032 (0.816, 1.193)	0.169
Cr/CysC ×100	73.929 ± 7.694	71.796 ± 10.545	0.113
CT body composition			
T12‐L4 SMVI	30.626 ± 2.545	30.965 ± 3.161	0.418
T12‐L4 SFI	52.689 (42.369, 72.617)	63.331 (35.515, 87.266)	0.288
T12‐L4 VFI	40.404 (22.265, 54.530)	45.137 (23.487, 69.607)	0.039*
T12‐L4 IMFI	2.670 (2.311, 3.202)	2.777 (2.232, 3.408)	0.612
T12‐L4 SMD	36.078 (32.900, 38.510)	36.558 (33.307, 39.516)	0.291
History			
Prior fragility fracture, *n* (%)	38/82 (46.3%)	47/104 (45.2%)	0.883
Baseline categories			
High sRANKL, *n* (%)	46/82 (56.1%)	53/104 (51.0%)	0.554
Low muscle strength, *n* (%)	77/82 (93.9%)	93/104 (89.4%)	0.307
Low muscle mass, *n* (%)	73/82 (89.0%)	90/104 (86.5%)	0.660
Sarcopenia, *n* (%)	69/82 (84.1%)	87/104 (83.7%)	1.000

*Note:* Continuous variables are shown as mean ± SD when approximately normally distributed in both comparison groups and as median (Q1, Q3) otherwise. Categorical variables are shown as *n/N* (%). The *p*‐values were derived from Welch *t*‐tests for approximately normally distributed continuous variables, Mann–Whitney *U*‐tests for nonnormally distributed continuous variables or Fisher exact tests, as appropriate.

Abbreviations: ASMI, appendicular skeletal muscle mass index; BDNF, brain‐derived neurotrophic factor; BMD, bone mineral density; BMI, body mass index; Cr/CysC ×100, creatinine/cystatin C × 100; CTX, C‐terminal telopeptide of type I collagen; DXA, dual‐energy x‐ray absorptiometry; HOMA‐IR, homeostasis model assessment of insulin resistance; hs‐CRP, high‐sensitivity C‐reactive protein; IGF‐1, insulin‐like growth factor 1; IL‐6, interleukin‐6; IMFI, intermuscular fat volume index; OPG, osteoprotegerin; P1NP, procollagen type I N‐terminal propeptide; P3NP, procollagen type III N‐terminal peptide; SFI, subcutaneous fat volume index; SMD, skeletal muscle density; SMVI, skeletal muscle volume index; sRANKL, soluble receptor activator of nuclear factor‐κB ligand; TNF‐α, tumour necrosis factor alpha; VFI, visceral fat volume index.

* *p* < 0.05.

** *p* < 0.01.

*** *p* < 0.001.

There were no significant between‐group differences in body mass index, lumbar spine or femoral neck BMD, whole‐body ASMI, grip strength, gait speed, chair‐stand performance, baseline low muscle strength, low muscle mass or sarcopenia prevalence (all *p* > 0.05). A limited number of secondary baseline variables showed modest between‐group differences, including vitamin D supplementation, brain‐derived neurotrophic factor, procollagen type III N‐terminal peptide, tumour necrosis factor alpha and T12‐L4 VFI. Follow‐up completion rates were similar in the two groups. Additional characteristics potentially related to treatment selection are summarized in Table S1. A history of bisphosphonate intolerance or inadequate response was more frequent in the denosumab group than in the zoledronic acid group (26.8% vs. 14.4%, *p* = 0.042), and administration preference differed between groups (*p* = 0.001), whereas anticipated treatment continuity was similar between groups (87.8% vs. 82.7%, *p* = 0.411).

The overall clinical trajectory favoured denosumab and is summarized in Figure 2. Functional differences were evident by 12 months and remained greater with denosumab than with zoledronic acid throughout follow‐up. At 12 months, grip strength increased by 1.45 ± 0.51 kg in the denosumab group versus 0.66 ± 0.34 kg in the zoledronic acid group (between‐group difference, 0.79; *p* < 0.001). Gait speed increased by 0.041 ± 0.013 m/s versus 0.017 ± 0.011 m/s (difference, 0.024; *p* < 0.001), and 5‐times chair stand time decreased by 0.82 ± 0.27 s versus 0.32 ± 0.18 s (difference, −0.50; *p* < 0.001). These between‐group differences increased over time and remained clear at 36 months. At that time point, grip strength had improved by 2.52 ± 0.63 kg in the denosumab group versus 0.99 ± 0.45 kg in the zoledronic acid group (difference, 1.53; *p* < 0.001), gait speed by 0.070 ± 0.017 m/s versus 0.027 ± 0.012 m/s (difference, 0.043; *p* < 0.001) and chair‐stand time by −1.47 s (−1.63, −1.34) versus −0.63 s (−0.82, −0.50) (difference, −0.86; *p* < 0.001) (Table 2 and Figure 2). Linear mixed‐effects models showed significant group‐by‐time interactions for all three primary functional outcomes (all *p* for interaction < 0.001), consistent with greater longitudinal functional improvement in the denosumab group.

**FIGURE 2:**
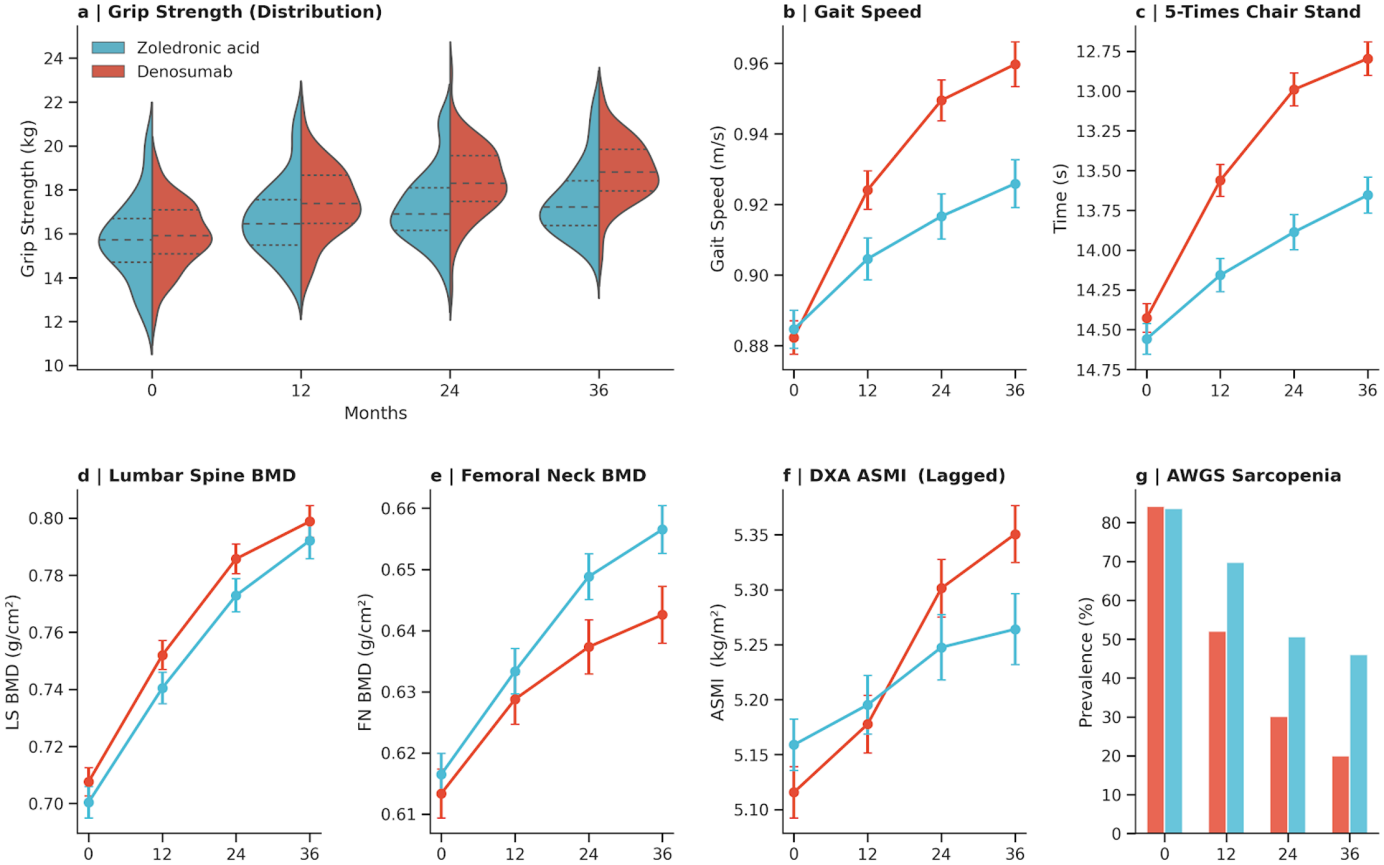
Longitudinal clinical, densitometric and study‐defined sarcopenia outcomes over 3 years. (A) Violin plots of observed grip‐strength distributions at baseline and at 12, 24 and 36 months in the denosumab and zoledronic acid groups. (B) Longitudinal trajectories of observed gait speed over follow‐up. (C) Longitudinal trajectories of observed 5‐times chair‐stand performance over follow‐up. (D) Longitudinal trajectories of lumbar spine BMD. (E) Longitudinal trajectories of femoral neck BMD. (F) Longitudinal trajectories of whole‐body DXA‐derived ASMI, showing delayed between‐group separation relative to the functional outcomes. (G) Prevalence of AWGS 2025‐informed, study‐defined sarcopenia across follow‐up visits. Violin plots display the observed distributions, with inner quartile markings. For the longitudinal trajectory panels, points represent observed means and error bars indicate standard errors of the mean.

**TABLE 2:** Longitudinal changes from baseline in functional performance, densitometric measures, biologic markers and CT‐derived body composition outcomes.

Variable	12 months			24 months			36 months			*P* for group × time
	Deno	ZA	*P*	Deno	ZA	*P*	Deno	ZA	*P*	
DXA										
Lumbar spine BMD, g/cm^2^	0.041 ± 0.008	0.032 ± 0.009	< 0.001***	0.070 (0.065, 0.076)	0.055 (0.051, 0.062)	< 0.001***	0.080 ± 0.007	0.071 ± 0.008	< 0.001***	< 0.001***
Femoral neck BMD, g/cm^2^	0.013 ± 0.005	0.013 ± 0.006	0.994	0.021 ± 0.007	0.024 ± 0.006	0.004**	0.026 ± 0.007	0.030 ± 0.007	< 0.001***	0.002**
Whole‐body DXA ASMI, kg/m^2^	0.056 ± 0.054	0.043 ± 0.051	0.119	0.161 ± 0.054	0.076 ± 0.054	< 0.001***	0.192 ± 0.059	0.089 ± 0.050	< 0.001***	< 0.001***
Function										
Grip strength, kg	1.45 ± 0.51	0.66 ± 0.34	< 0.001***	2.22 ± 0.63	0.99 ± 0.39	< 0.001***	2.52 ± 0.63	0.99 ± 0.45	< 0.001***	< 0.001***
Gait speed, m/s	0.041 ± 0.013	0.017 ± 0.011	< 0.001***	0.062 ± 0.016	0.024 ± 0.012	< 0.001***	0.070 ± 0.017	0.027 ± 0.012	< 0.001***	< 0.001***
5‐times chair stand, s	−0.82 ± 0.27	−0.32 ± 0.18	< 0.001***	−1.34 ± 0.33	−0.53 ± 0.25	< 0.001***	−1.47 (−1.63, −1.34)	−0.63 (−0.82, −0.50)	< 0.001***	< 0.001***
RANKL/OPG and bone turnover										
sRANKL, pmol/L	−0.370 (−0.394, −0.341)	−0.143 (−0.180, −0.113)	< 0.001***	−0.506 ± 0.067	−0.221 ± 0.055	< 0.001***	−0.554 ± 0.075	−0.225 ± 0.055	< 0.001***	< 0.001***
OPG, pmol/L	0.174 ± 0.072	0.070 ± 0.068	< 0.001***	0.271 ± 0.077	0.102 ± 0.072	< 0.001***	0.312 ± 0.076	0.122 ± 0.073	< 0.001***	< 0.001***
OPG/sRANKL ratio	2.503 (2.053, 3.429)	0.807 (0.549, 0.994)	< 0.001***	4.561 (3.697, 5.830)	1.419 (1.112, 1.770)	< 0.001***	5.677 (4.437, 7.067)	1.448 (1.157, 1.995)	< 0.001***	< 0.001***
CTX, ng/mL	−0.436 ± 0.032	−0.261 ± 0.033	< 0.001***	−0.481 ± 0.044	−0.319 ± 0.030	< 0.001***	−0.516 (−0.534, −0.484)	−0.330 (−0.344, −0.307)	< 0.001***	< 0.001***
P1NP, ng/mL	−28.41 ± 2.77	−17.59 ± 2.62	< 0.001***	−30.09 (−31.35, −28.07)	−19.55 (−21.37, −18.30)	< 0.001***	−30.82 (−32.27, −29.17)	−20.31 (−22.28, −18.39)	< 0.001***	< 0.001***
Myokines/laboratory										
Myostatin, ng/mL	−0.225 ± 0.052	−0.104 ± 0.051	< 0.001***	−0.346 ± 0.050	−0.160 ± 0.057	< 0.001***	−0.383 ± 0.050	−0.160 ± 0.056	< 0.001***	< 0.001***
Follistatin, ng/mL	0.029 ± 0.020	0.019 ± 0.020	0.002**	0.053 ± 0.019	0.026 ± 0.022	< 0.001***	0.064 ± 0.022	0.031 ± 0.020	< 0.001***	< 0.001***
Follistatin/myostatin ratio	0.030 ± 0.008	0.015 ± 0.008	< 0.001***	0.051 ± 0.011	0.023 ± 0.009	< 0.001***	0.059 ± 0.012	0.025 ± 0.009	< 0.001***	< 0.001***
Irisin, ng/mL	0.243 ± 0.065	0.110 ± 0.063	< 0.001***	0.417 ± 0.074	0.157 ± 0.075	< 0.001***	0.481 ± 0.073	0.186 ± 0.064	< 0.001***	< 0.001***
BDNF, ng/mL	0.36 ± 0.38	0.31 ± 0.42	0.371	0.45 ± 0.30	0.44 ± 0.34	0.726	0.61 ± 0.36	0.54 ± 0.34	0.224	0.740
P3NP, μg/L	0.049 ± 0.051	0.033 ± 0.052	0.050	0.084 ± 0.050	0.063 ± 0.053	0.015*	0.127 ± 0.049	0.084 ± 0.057	< 0.001***	< 0.001***
IGF‐1, ng/mL	2.6 ± 1.9	1.8 ± 1.9	0.008**	4.3 ± 1.6	3.8 ± 1.9	0.091	4.5 (3.4, 6.1)	4.2 (2.9, 5.7)	0.123	0.131
Inflammation										
hs‐CRP, mg/L	−0.176 ± 0.130	−0.105 ± 0.125	< 0.001***	−0.278 ± 0.127	−0.168 ± 0.129	< 0.001***	−0.352 ± 0.112	−0.188 ± 0.116	< 0.001***	< 0.001***
IL‐6, pg./mL	−0.189 ± 0.135	−0.148 ± 0.120	0.040*	−0.288 ± 0.126	−0.164 ± 0.125	< 0.001***	−0.352 ± 0.129	−0.207 ± 0.136	< 0.001***	< 0.001***
TNF‐α, pg./mL	−0.066 ± 0.050	−0.034 ± 0.048	< 0.001***	−0.117 (−0.148, −0.088)	−0.049 (−0.085, −0.019)	< 0.001***	−0.144 ± 0.050	−0.064 ± 0.049	< 0.001***	< 0.001***
Metabolic										
Fasting glucose, mmol/L	−0.071 ± 0.095	−0.019 ± 0.091	< 0.001***	−0.114 ± 0.103	−0.041 ± 0.106	< 0.001***	−0.130 ± 0.105	−0.052 ± 0.104	< 0.001***	< 0.001***
Fasting insulin, uIU/mL	−0.879 ± 0.446	−0.231 ± 0.439	< 0.001***	−1.395 ± 0.447	−0.537 ± 0.416	< 0.001***	−1.638 ± 0.453	−0.487 ± 0.452	< 0.001***	< 0.001***
HOMA‐IR	−0.235 ± 0.112	−0.064 ± 0.115	< 0.001***	−0.371 ± 0.114	−0.145 ± 0.114	< 0.001***	−0.433 ± 0.114	−0.136 ± 0.124	< 0.001***	< 0.001***
Laboratory										
Creatinine, mg/dL	0.002 ± 0.021	0.000 ± 0.023	0.626	0.007 ± 0.018	0.001 ± 0.021	0.053	0.004 ± 0.021	0.000 ± 0.019	0.239	0.591
Cystatin C, mg/L	0.001 ± 0.022	−0.002 ± 0.018	0.284	0.002 ± 0.022	−0.000 ± 0.020	0.517	0.004 ± 0.022	−0.001 ± 0.019	0.204	0.647
Cr/CysC ×100	0.060 ± 2.962	0.163 ± 2.910	0.822	0.476 ± 2.991	0.146 ± 2.921	0.503	0.260 ± 3.032	0.126 ± 2.974	0.801	0.938
CT body composition										
T12‐L4 SMVI	1.185 ± 0.498	0.453 ± 0.487	< 0.001***	1.981 (1.608, 2.570)	0.611 (0.309, 1.028)	< 0.001***	2.351 (1.959, 3.012)	0.655 (0.419, 0.987)	< 0.001***	< 0.001***
T12‐L4 SFI	−0.051 ± 1.195	−0.057 ± 1.350	0.975	0.241 ± 1.535	0.073 ± 1.322	0.490	0.034 ± 1.712	−0.174 ± 1.388	0.469	0.909
T12‐L4 VFI	−0.779 ± 1.309	−0.436 ± 1.198	0.087	−1.012 ± 1.294	−0.206 ± 1.215	< 0.001***	−1.319 ± 1.176	−0.506 ± 1.234	< 0.001***	0.002**
T12‐L4 IMFI	−0.101 ± 0.081	−0.036 ± 0.087	< 0.001***	−0.166 ± 0.095	−0.036 ± 0.081	< 0.001***	−0.220 ± 0.094	−0.039 ± 0.080	< 0.001***	< 0.001***
T12‐L4 SMD	1.468 ± 0.604	0.466 ± 0.487	< 0.001***	2.530 (2.035, 3.082)	0.986 (0.548, 1.415)	< 0.001***	3.022 ± 1.103	0.937 ± 0.671	< 0.001***	< 0.001***

*Note:* Values are change‐from‐baseline mean ± SD when approximately normally distributed in both comparison groups and median (Q1, Q3) otherwise, among participants with available data at each follow‐up visit. The last column shows the global *p*‐value for the group × time interaction from linear mixed‐effects models. Pointwise between‐group *p*‐values were derived from Welch *t*‐tests for approximately normally distributed change scores and Mann–Whitney *U* tests otherwise. Continuous outcomes were additionally evaluated using linear mixed‐effects models with fixed effects for group, categorical follow‐up visit and group × time and a subject‐specific random intercept.

Abbreviations: ASMI, appendicular skeletal muscle mass index; BDNF, brain‐derived neurotrophic factor; Cr/CysC ×100, creatinine/cystatin C × 100; CTX, C‐terminal telopeptide of type I collagen; DXA, dual‐energy x‐ray absorptiometry; HOMA‐IR, homeostasis model assessment of insulin resistance; hs‐CRP, high‐sensitivity C‐reactive protein; IGF‐1, insulin‐like growth factor 1; IL‐6, interleukin‐6; IMFI, intermuscular fat volume index; OPG, osteoprotegerin; P1NP, procollagen type I N‐terminal propeptide; P3NP, procollagen type III N‐terminal peptide; SFI, subcutaneous fat volume index; SMD, skeletal muscle density; SMVI, skeletal muscle volume index; sRANKL, soluble receptor activator of nuclear factor‐κB ligand; TNF‐α, tumour necrosis factor alpha; VFI, visceral fat volume index; ZA, zoledronic acid.

* *p* < 0.05.

** *p* < 0.01.

*** *p* < 0.001.

DXA findings followed a later course. Whole‐body ASMI did not differ significantly between groups at 12 months (0.056 ± 0.054 vs. 0.043 ± 0.051 kg/m^2^; *p* = 0.119), but a between‐group difference became apparent at 24 months (0.161 ± 0.054 vs. 0.076 ± 0.054 kg/m^2^; difference, 0.085; *p* < 0.001) and remained present at 36 months (0.192 ± 0.059 vs. 0.089 ± 0.050 kg/m^2^; difference, 0.103; *p* < 0.001). Lumbar spine BMD increased more in the denosumab group at every follow‐up visit (all *p* < 0.001). In contrast, femoral neck BMD did not differ at 12 months and showed slightly greater gains in the zoledronic acid group at 24 and 36 months (*p* = 0.004 and *p* < 0.001, respectively). Taken together, functional improvement appeared earlier, whereas separation in DXA‐defined muscle mass emerged later. In the linear mixed‐effects models, the group‐by‐time interaction was significant for lumbar spine BMD, femoral neck BMD and ASMI.

In exploratory analyses, changes in lumbar spine BMD were positively correlated with changes in grip strength in the pooled cohort at 12, 24 and 36 months (*r* = 0.347, 0.471 and 0.417, respectively; all *p* < 0.001). However, these associations were weak and nonsignificant within each treatment group, and lumbar spine BMD change was not independently associated with grip strength change after adjustment for treatment group and age (all *p* ≥ 0.622). Femoral neck BMD change showed no consistent association with grip strength change in within‐group or adjusted analyses (Table S2).

At baseline, 85 participants (45.7%) were aged 50–64 years and 101 (54.3%) were aged ≥ 65 years. In exploratory age‐stratified analyses at 36 months, the between‐group differences in grip strength, gait speed and 5‐times chair stand time were similar in direction across both age strata. There was no evidence of treatment‐by‐age‐stratum interaction for grip strength (*p* = 0.705), gait speed (*p* = 0.770) or 5‐times chair stand time (*p* = 0.341) (Table S3).

Study‐defined categorical sarcopenia outcomes under the AWGS 2025‐informed framework followed the same pattern. Low muscle strength was less common in the denosumab group at 12 months (61.8% vs. 79.1%, *p* = 0.017), 24 months (38.8% vs. 71.2%, *p* < 0.001) and 36 months (26.7% vs. 69.6%, *p* < 0.001). By contrast, the prevalence of low muscle mass did not differ significantly between groups at any follow‐up visit. Sarcopenia prevalence was lower with denosumab at 12 months (52.1% vs. 69.8%, *p* = 0.033), 24 months (30.2% vs. 50.7%, *p* = 0.016) and 36 months (20.0% vs. 46.0%, *p* = 0.003). Among participants with sarcopenia at baseline, remission was more frequent in the denosumab group at 12 months (36.7% vs. 15.5%, *p* = 0.008), 24 months (63.5% vs. 37.3%, *p* = 0.008) and 36 months (75.0% vs. 37.0%, *p* < 0.001). These findings support a clinically coherent pattern in which earlier gains in muscle function were followed by a lower burden of study‐defined sarcopenia under the AWGS 2025‐informed framework and higher remission rates (Table 3).

**TABLE 3:** Categorical sarcopenia outcomes, biologic responder profiles, CT remodeling according to responder status and exploratory mediation analyses.

Outcome	Time point/metric	Denosumab or high responder	Zoledronic acid or low responder	*p*‐value/summary
A. AWGS 2025‐informed, study‐defined sarcopenia outcomes and cumulative clinical events				
Low muscle strength	12 months	47/76 (61.8%)	72/91 (79.1%)	0.017*
Low muscle strength	24 months	26/67 (38.8%)	57/80 (71.2%)	< 0.001***
Low muscle strength	36 months	16/60 (26.7%)	48/69 (69.6%)	< 0.001***
Low muscle mass	12 months	58/73 (79.5%)	70/86 (81.4%)	0.842
Low muscle mass	24 months	39/63 (61.9%)	49/75 (65.3%)	0.724
Low muscle mass	36 months	34/55 (61.8%)	40/63 (63.5%)	1.000
Sarcopenia	12 months	38/73 (52.1%)	60/86 (69.8%)	0.033*
Sarcopenia	24 months	19/63 (30.2%)	38/75 (50.7%)	0.016*
Sarcopenia	36 months	11/55 (20.0%)	29/63 (46.0%)	0.003**
Sarcopenia remission	12 months	22/60 (36.7%)	11/71 (15.5%)	0.008**
Sarcopenia remission	24 months	33/52 (63.5%)	22/59 (37.3%)	0.008**
Sarcopenia remission	36 months	33/44 (75.0%)	17/46 (37.0%)	< 0.001***
≥ 1 fall, cumulative	12 months	11/76 (14.5%)	16/91 (17.6%)	0.675
≥ 1 fall, cumulative	24 months	15/67 (22.4%)	21/80 (26.2%)	0.701
≥ 1 fall, cumulative	36 months	16/60 (26.7%)	25/69 (36.2%)	0.262
New fragility fracture, cumulative	12 months	2/76 (2.6%)	5/91 (5.5%)	0.457
New fragility fracture, cumulative	24 months	3/67 (4.5%)	6/80 (7.5%)	0.510
New fragility fracture, cumulative	36 months	3/60 (5.0%)	7/69 (10.1%)	0.337
B. Biologic responder analysis within the denosumab group				
Baseline high‐sRANKL rate	High vs. low responder	18/38 (47.4%)	22/38 (57.9%)	0.491
12‐mo ΔOPG/sRANKL	High vs. low responder	3.464 (3.012, 4.247)	2.052 (1.652, 2.282)	< 0.001***
12‐mo ΔMyostatin	High vs. low responder	−0.236 ± 0.047	−0.215 ± 0.055	0.079
12‐mo ΔIrisin	High vs. low responder	0.263 ± 0.063	0.222 ± 0.061	0.004**
36‐mo ΔGrip	High vs. low responder	2.802 ± 0.652	2.215 ± 0.438	< 0.001***
36‐mo ΔASMI	High vs. low responder	0.216 (0.180, 0.242)	0.164 (0.139, 0.204)	0.002**
36‐mo ΔHOMA‐IR	High vs. low responder	−0.459 ± 0.101	−0.406 ± 0.122	0.072
36‐mo remission	High vs. low responder	22/24 (91.7%)	11/20 (55.0%)	0.012*
C. CT remodelling according to biologic responder status				
12 months T12‐L4 SMVI	High vs. low responder	1.334 ± 0.500	1.037 ± 0.455	0.009**
12 months T12‐L4 SFI	High vs. low responder	−0.039 ± 1.263	−0.062 ± 1.140	0.935
12 months T12‐L4 VFI	High vs. low responder	−0.822 ± 1.335	−0.735 ± 1.299	0.776
12 months T12‐L4 IMFI	High vs. low responder	−0.123 ± 0.079	−0.079 ± 0.078	0.019*
12 months T12‐L4 SMD	High vs. low responder	1.614 ± 0.641	1.322 ± 0.535	0.037*
24 months T12‐L4 SMVI	High vs. low responder	2.502 ± 0.739	1.694 ± 0.391	< 0.001***
24 months T12‐L4 SFI	High vs. low responder	0.420 ± 1.818	0.032 ± 1.115	0.298
24 months T12‐L4 VFI	High vs. low responder	−1.176 (−1.979, −0.629)	−1.097 (−1.674, −0.214)	0.767
24 months T12‐L4 IMFI	High vs. low responder	−0.184 ± 0.100	−0.145 ± 0.086	0.097
24 months T12‐L4 SMD	High vs. low responder	2.765 (2.389, 3.497)	2.125 (1.781, 2.596)	< 0.001***
36 months T12‐L4 SMVI	High vs. low responder	2.816 ± 0.861	2.199 ± 0.524	0.002**
36 months T12‐L4 SFI	High vs. low responder	−0.188 ± 1.616	0.273 ± 1.810	0.321
36 months T12‐L4 VFI	High vs. low responder	−1.429 ± 1.099	−1.201 ± 1.264	0.475
36 months T12‐L4 IMFI	High vs. low responder	−0.253 ± 0.091	−0.184 ± 0.087	0.005**
36 months T12‐L4 SMD	High vs. low responder	3.132 (2.761, 4.173)	2.716 (2.311, 3.189)	0.017*
D. Exploratory mediation analysis of the OPG/sRANKL axis				
Treatment → 12‐mo ΔOPG/sRANKL → 36‐mo ΔGrip	Indirect effect (95% CI)	0.566 (0.39, 0.74)	Proportion mediated: 37.0%	
Treatment → 12‐mo ΔOPG/sRANKL → 36‐mo ΔGrip	Direct effect (95% CI)	0.964 (0.723, 1.205)	Total effect (95% CI): 1.530 (1.342 to 1.719)	
Treatment → 12‐mo ΔOPG/sRANKL → 36‐mo ΔCT SMVI	Indirect effect (95% CI)	0.747 (0.50, 1.02)	Proportion mediated: 39.9%	
Treatment → 12‐mo ΔOPG/sRANKL → 36‐mo ΔCT SMVI	Direct effect (95% CI)	1.126 (0.836, 1.417)	Total effect (95% CI): 1.873 (1.637 to 2.109)	

*Note:* Panel A shows the categorical outcomes and cumulative clinical events, Panel B the biologic responder analyses within the denosumab group, Panel C the CT remodelling according to biologic responder status and Panel D the exploratory mediation analyses of the OPG/sRANKL axis. Unless otherwise indicated, continuous data are shown as mean ± SD when approximately normally distributed in both comparison groups and as median (Q1, Q3) otherwise and categorical data as *n/N* (%). Indirect, direct and total effects are reported with 95% CIs.

Abbreviations: ASMI, appendicular skeletal muscle mass index; AWGS, Asian Working Group for Sarcopenia; CI, confidence interval; CT, computed tomography; CTX, C‐terminal telopeptide of type I collagen; DXA, dual‐energy x‐ray absorptiometry; HOMA‐IR, homeostasis model assessment of insulin resistance; IMFI, intermuscular fat volume index; OPG, osteoprotegerin; P1NP, procollagen type I N‐terminal propeptide; SFI, subcutaneous fat volume index; SMD, skeletal muscle density; SMVI, skeletal muscle volume index; sRANKL, soluble receptor activator of nuclear factor‐κB ligand; VFI, visceral fat volume index; ZA, zoledronic acid.

* *p* < 0.05.

** *p* < 0.01.

*** *p* < 0.001.

The structural counterpart to this clinical pattern is shown in Figure 3. CT‐based body composition analyses demonstrated coordinated remodelling across muscle and adipose compartments rather than change in a single isolated parameter. At 12 months, denosumab was associated with a greater increase in T12‐L4 SMVI than zoledronic acid (1.19 ± 0.50 vs. 0.45 ± 0.49; *p* < 0.001), a greater increase in SMD (1.47 ± 0.60 vs. 0.47 ± 0.49; *p* < 0.001) and a greater reduction in IMFI (−0.10 ± 0.08 vs. − 0.04 ± 0.09; *p* < 0.001). VFI showed a nonsignificant trend at 12 months, whereas SFI did not differ between groups. By 24 and 36 months, between‐group differences remained pronounced for SMVI, IMFI, and SMD, and the reduction in VFI became significant, whereas SFI remained similar between groups (Table 2 and Figure 3). Linear mixed‐effects models likewise showed significant group‐by‐time interactions for SMVI, VFI, IMFI and SMD, whereas SFI showed no significant interaction, supporting a more favourable longitudinal remodelling pattern with denosumab.

**FIGURE 3:**
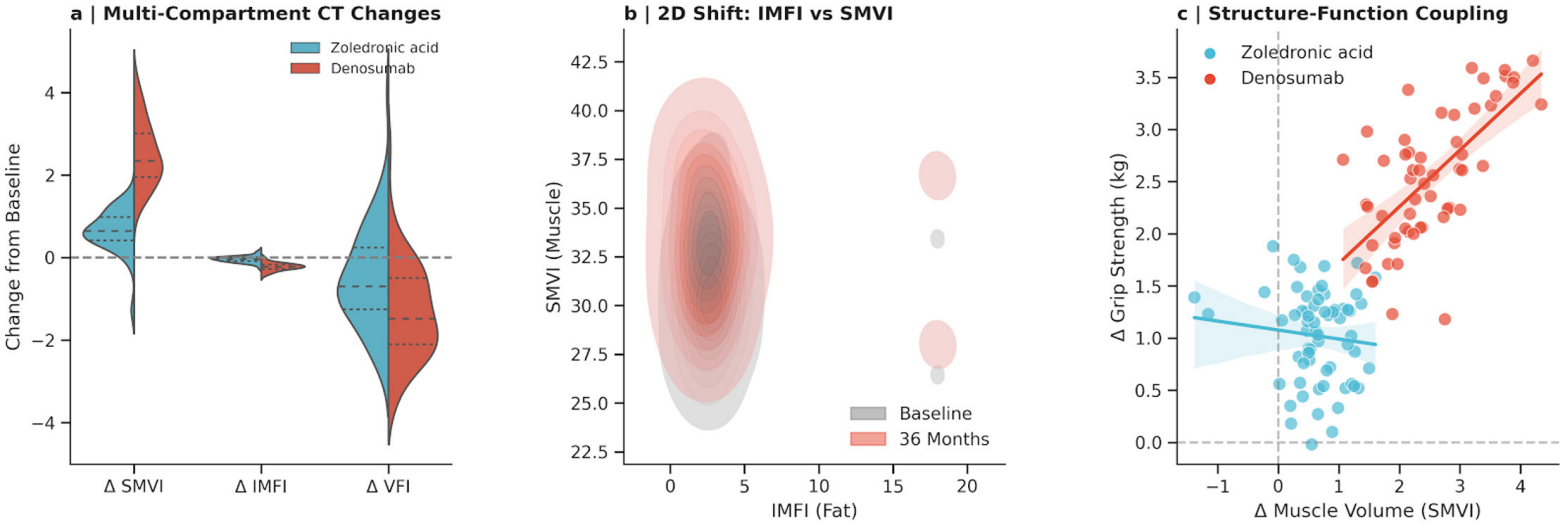
CT‐defined structural remodelling and structure–function coupling. (A) Violin plots of 36‐month change‐from‐baseline distributions for T12‐L4 SMVI, IMFI and VFI in the denosumab and zoledronic acid groups. (B) Two‐dimensional density shift in IMFI and SMVI from baseline to 36 months, illustrating coordinated remodelling across muscle and adipose compartments. (C) Scatterplot of 36‐month change in grip strength versus 36‐month change in SMVI, with separate fitted lines for the denosumab and zoledronic acid groups.

The biologic basis for these downstream changes is shown in Figure 4. Denosumab produced a greater separation in the RANKL/OPG axis, with a larger reduction in sRANKL, a greater increase in OPG and a substantially greater rise in the OPG/sRANKL ratio at every follow‐up time point. At 12 months, the increase in the OPG/sRANKL ratio was 2.503 (2.053, 3.429) in the denosumab group versus 0.807 (0.549, 0.994) in the zoledronic acid group (*p* < 0.001). This between‐group difference increased further at 24 months (4.561 [3.697, 5.830] vs. 1.419 [1.112, 1.770]; *p* < 0.001) and remained marked at 36 months (5.677 [4.437, 7.067] vs. 1.448 [1.157, 1.995]; *p* < 0.001). Bone‐turnover markers followed the same pattern, with greater reductions in CTX and P1NP throughout follow‐up (Table 2). Linear mixed‐effects models confirmed significant group‐by‐time interactions for sRANKL, OPG the OPG/sRANKL ratio, CTX and P1NP (all P for interaction < 0.001).

**FIGURE 4:**
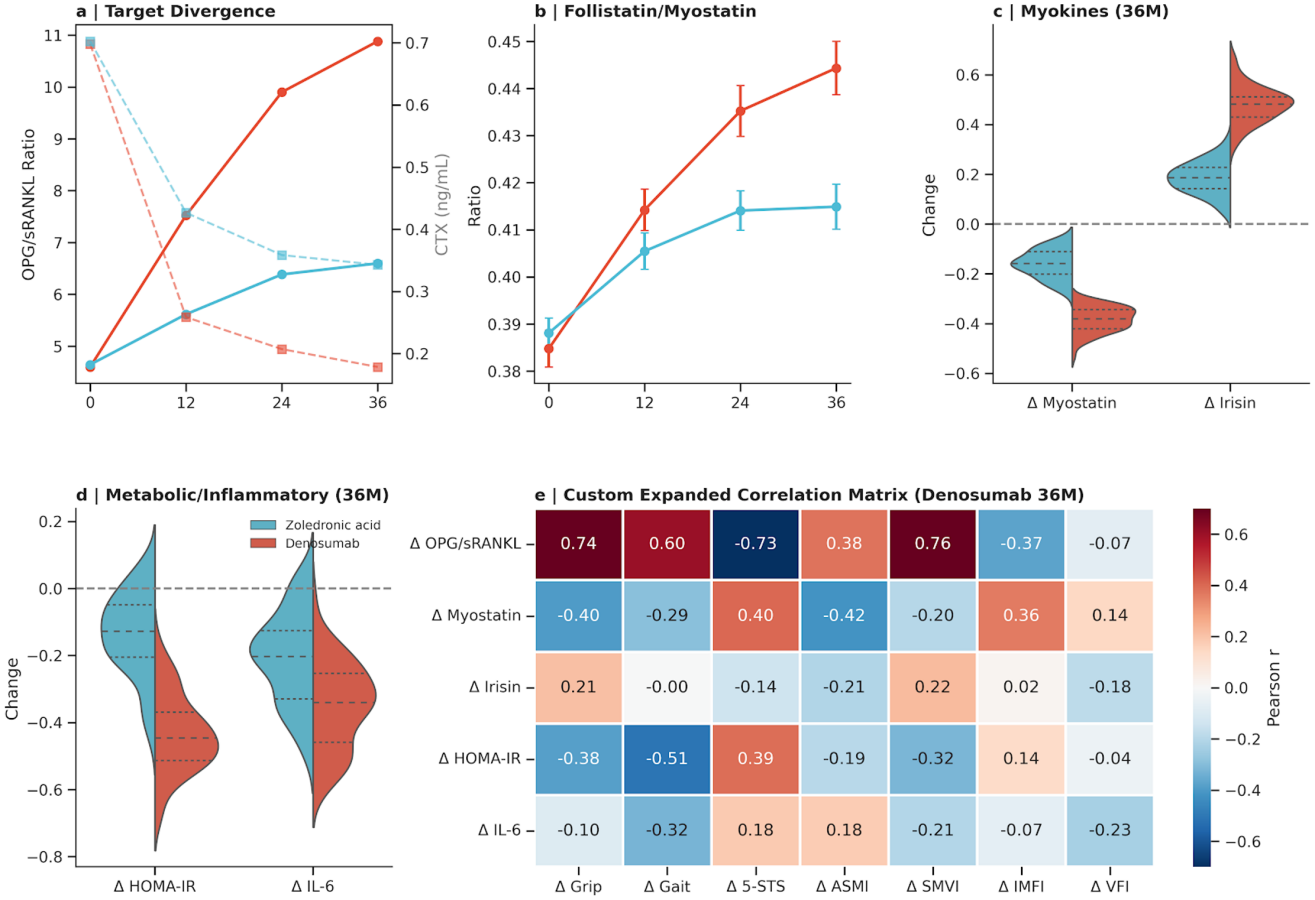
Biologic divergence in the OPG/sRANKL axis and representative downstream adaptations. (A) Longitudinal divergence in the OPG/sRANKL ratio and CTX over follow‐up, shown with dual y axes. (B) Longitudinal changes in the follistatin/myostatin ratio. (C) Violin plots of 36‐month changes in representative myokines, including myostatin and irisin. (D) Violin plots of 36‐month changes in representative metabolic and inflammatory markers, including HOMA‐IR and IL‐6. (E) Pearson correlation matrix at 36 months within denosumab‐treated participants, summarizing relationships between biologic changes and functional or CT‐derived structural outcomes. Violin plots display the observed distributions, with inner quartile markings. For the longitudinal trajectory panels, points represent observed means and error bars indicate standard errors of the mean, where shown.

In sensitivity analyses using adjusted linear mixed‐effects models that additionally controlled for age, baseline vitamin D supplementation and baseline variables that differed between groups, the main findings were materially unchanged for the primary functional outcomes, whole‐body ASMI, lumbar spine and femoral neck BMD, the OPG/sRANKL ratio and CT‐derived SMVI, IMFI, and SMD (Table S4). For VFI, adjusted between‐group differences remained significant at 24 and 36 months, although the overall adjusted group‐by‐time interaction was attenuated.

Regular physical activity, resistance exercise and physical therapy or rehabilitation did not differ significantly between treatment groups at baseline or at any follow‐up visit (all *p* ≥ 0.345). In additional linear mixed‐effects models incorporating these variables as time‐varying covariates, the between‐group differences in the primary functional outcomes were materially unchanged, and the group‐by‐time interactions remained significant for grip strength, gait speed and 5‐times chair stand time (all *p* < 0.001) (Tables S5 and S6).

In the propensity score‐weighted sensitivity analysis, covariate balance improved substantially after weighting, with the maximum absolute SMD decreasing from 0.446 to 0.110. The weighted between‐group differences at 36 months remained significant for grip strength (1.607 kg; 95% CI, 1.386–1.828; *p* < 0.001), gait speed (0.0455 m/s; 95% CI, 0.0403–0.0507; *p* < 0.001) and 5‐times chair stand time (−0.863 s; 95% CI, −0.953 to −0.772; *p* < 0.001), consistent with the primary analyses (Tables S7 and S8).

Myostatin showed a greater decline over follow‐up in the denosumab group, whereas irisin increased to a greater extent. BDNF did not differ significantly in longitudinal change at 12, 24 or 36 months (*p* = 0.371, 0.726 and 0.224, respectively). P3NP favoured denosumab at 24 and 36 months (*p* = 0.015 and *p* < 0.001, respectively), with a borderline difference at 12 months (*p* = 0.050). TNF‐α decreased more with denosumab at all follow‐up visits (all *p* < 0.001), and similar patterns were observed for hs‐CRP, IL‐6, fasting insulin, and HOMA‐IR. Among denosumab completers, the 12‐month change in the OPG/sRANKL ratio correlated with change in grip strength (*r* = 0.520, *p* < 0.001), gait speed (*r* = 0.364, *p* = 0.001), 5‐times chair stand time (*r* = −0.470, *p* < 0.001), and ASMI (*r* = 0.316, *p* = 0.006). Associations in the same direction were observed at later follow‐up, and the 36‐month correlation structure is summarized in Figure 4. Overall, the biologic separation associated with denosumab extended to muscle‐related, metabolic and inflammatory adaptation and was consistently linked to later clinical and imaging benefit (Table 2 and Figure 4).

Figure 5 addresses response heterogeneity within the denosumab group. High responders, defined prespecifically as participants with a 12‐month increase in the OPG/sRANKL ratio at or above the within‐group median, did not differ from low responders in baseline high‐sRANKL prevalence (47.4% vs. 57.9%, *p* = 0.491). They did, however, show a substantially greater 12‐month rise in the OPG/sRANKL ratio (3.464 [3.012, 4.247] vs. 2.052 [1.652, 2.282]; *p* < 0.001). By 36 months, high responders had greater gains in grip strength (2.80 ± 0.65 vs. 2.21 ± 0.44 kg; *p* < 0.001) and CT‐defined SMVI (2.816 ± 0.861 vs. 2.199 ± 0.524; *p* = 0.002) than low responders. A modestly greater gain in ASMI was also observed [0.216 (0.180, 0.242) vs. 0.164 (0.139, 0.204) kg/m^2^; *p* = 0.002], whereas the greater reduction in HOMA‐IR remained directionally favourable but did not reach statistical significance (*p* = 0.072). High responders also had a markedly higher sarcopenia remission rate at 36 months (91.7% vs. 55.0%, *p* = 0.012) (Table 3 and Figure 5).

**FIGURE 5:**
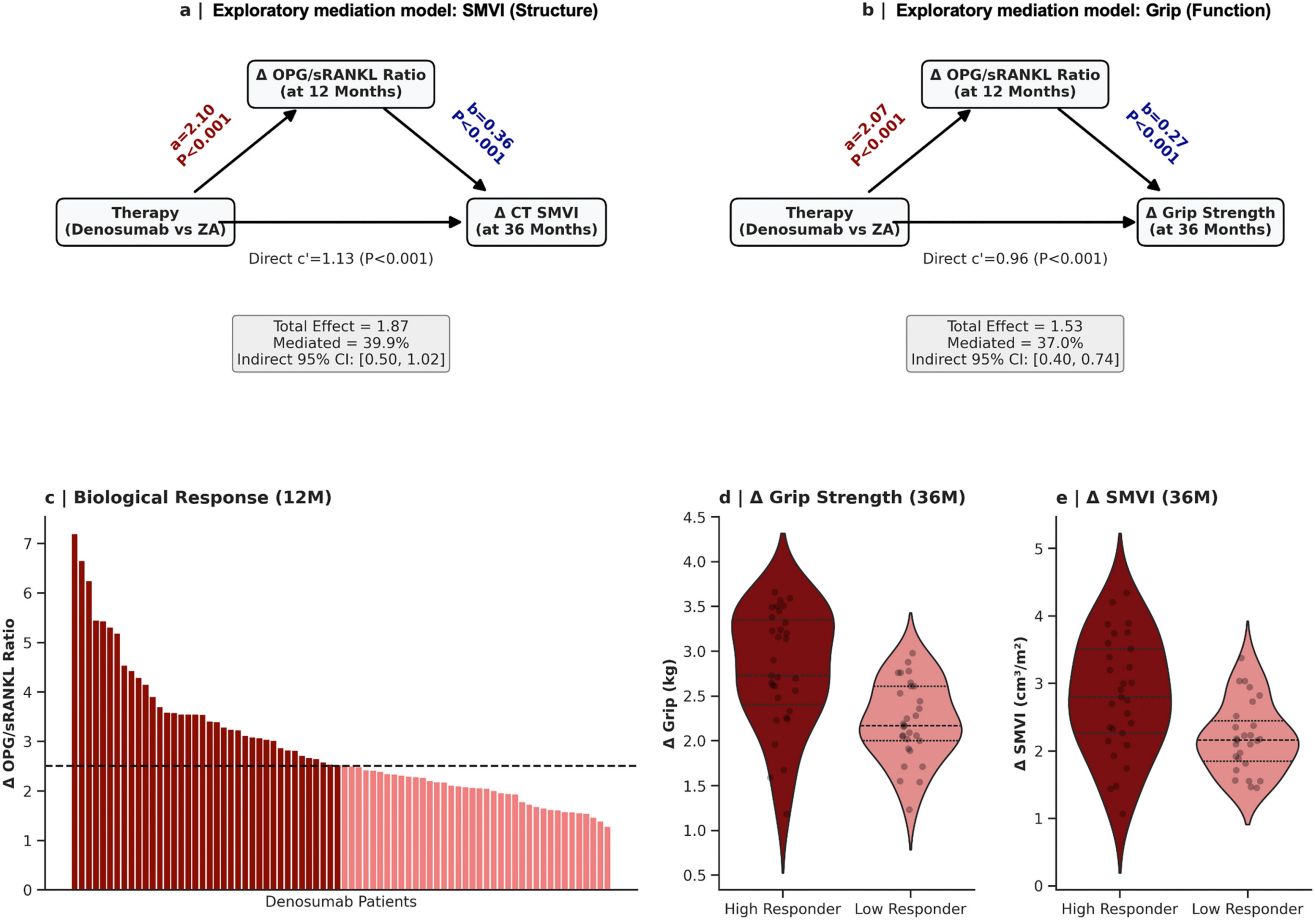
Biologic responder heterogeneity and mediation of representative functional and structural outcomes. (A) Exploratory mediation model illustrating the association between treatment group and 36‐month CT‐defined SMVI gain in relation to the 12‐month change in the OPG/sRANKL ratio. (B) Exploratory mediation model illustrating the association between treatment group and 36‐month grip‐strength gain in relation to the 12‐month change in the OPG/sRANKL ratio. Path coefficients, direct effects, total effects and proportion mediated are displayed within the panels. (C) Waterfall‐style display of the 12‐month biologic response among denosumab‐treated participants; the dashed horizontal line indicates the within‐group median used to classify biologic high and low responders. (D) Violin plots of 36‐month grip‐strength gain according to biologic responder status. (E) Violin plots of 36‐month CT‐defined SMVI gain according to biologic responder status.

A similar pattern was seen for CT‐based remodelling. At 12 months, high responders showed larger increases in SMVI (*p* = 0.009) and SMD (*p* = 0.037) and a greater reduction in IMFI (*p* = 0.019). At 24 months, the largest between‐group differences were observed for SMVI and SMD (both *p* < 0.001). At 36 months, high responders maintained advantages in SMVI (*p* = 0.002), IMFI (*p* = 0.005) and SMD (*p* = 0.017), whereas SFI and VFI did not differ consistently between responder strata. Exploratory mediation analyses were also consistent with the OPG/sRANKL axis as an early biologic correlate of treatment response. In the model using 36‐month grip‐strength gain as the outcome, the 12‐month increase in the OPG/sRANKL ratio showed a pattern consistent with partial mediation of the association between treatment and subsequent improvement in grip strength, with an indirect effect of 0.566 (95% CI, 0.39–0.74), accounting for 37.0% of the total treatment effect. The direct effect remained significant at 0.964 (95% CI, 0.723–1.205; *p* < 0.001), supporting partial mediation.

Grip strength and CT‐defined SMVI were selected as representative functional and structural response end points, respectively, because both showed clear longitudinal between‐group separation and were further enhanced in biologic high responders. A similar mediation pattern was observed for 36‐month CT‐defined SMVI gain, with an indirect effect of 0.747 (95% CI, 0.50–1.02), accounting for 39.9% of the total treatment effect, together with a significant residual direct effect of 1.126 (95% CI, 0.836–1.417; *p* < 0.001). These findings support an association between early biologic activation and later functional improvement and structural remodelling, with a pattern statistically consistent with partial mediation (Table 3 and Figure 5).

Event outcomes were directionally favourable but modest. Falls and new fragility fractures did not differ significantly between groups at 12, 24 or 36 months (Table 3). Overall, the data support a coherent sequence in which denosumab was associated first with stronger biologic pathway activation, followed by earlier gains in muscle strength and physical performance, coordinated CT‐defined remodelling, later separation in DXA‐derived muscle mass and, ultimately, greater improvement in study‐defined sarcopenia outcomes under an AWGS 2025‐informed framework.

## 4 Discussion

Our findings support a staged treatment pattern in which denosumab‐related biologic divergence preceded functional improvement, followed by CT‐defined remodelling and only later separation in DXA‐defined muscle mass. Comparative clinical studies have reported favourable effects of denosumab on grip strength or chair‐rise performance relative to conventional comparators [23, 24]. More recent clinical studies in Asian populations have also described improvements in strength, gait‐related function or related muscle indices after denosumab exposure [25, 26]. Systematic reviews likewise suggest that the most consistent muscle‐related benefits of denosumab are observed in strength and physical performance rather than in appendicular lean mass per se [20].

The clinical importance of this staged response extends beyond densitometry because better physical function is itself patient‐relevant in osteosarcopenia. Pooled trial data have shown that denosumab can be associated with lower fall incidence, supporting the idea that functional improvement may translate into clinically meaningful benefit even when changes in lean mass are modest [27]. More broadly, osteosarcopenia has been associated with higher fall and fracture risk, poorer activities of daily living and greater frailty burden than isolated osteoporosis or sarcopenia alone [3, 28, 29, 30].

Our CT results substantially strengthen this interpretation. Greater gains in skeletal muscle volume and density together with lower intermuscular fat favour an improvement in muscle quality rather than a nonspecific shift in body composition. Contemporary reviews of osteosarcopenia increasingly emphasize that bone, muscle and adipose tissue communicate through shared biochemical networks, making coordinated remodelling across these compartments biologically plausible [31, 32, 33, 34]. Recent translational syntheses further support the view that ectopic fat infiltration and impaired muscle quality are central intermediates linking endocrine and inflammatory signalling to later functional decline [35, 36].

The biomarker profile in our cohort fits this framework. Larger reductions in myostatin, inflammatory markers and insulin‐resistance indices in the denosumab group are consistent with the concept that RANKL blockade acts within a wider musculoskeletal‐metabolic axis rather than through bone alone [31, 36]. This interpretation is also aligned with newer reviews of sarcopenia within the musculoskeletal system and meta‐analytic evidence showing strong epidemiologic coupling between sarcopenia and osteoporosis [37, 38].

The responder analysis adds a translational layer. Patients with larger early OPG/sRANKL shifts experienced greater later functional and structural benefit; however, this pattern should be interpreted as an association rather than as evidence that early biomarker change directly determined subsequent treatment response. Biologic responsiveness may therefore represent one correlate of heterogeneity in treatment effect rather than a confirmed mechanistic intermediary. Longitudinal cohort data have shown that the association between osteosarcopenia and frailty is directionally adverse but heterogeneous across community‐based populations [28, 39, 40]. In parallel, meta‐analytic synthesis indicates that coexistence of bone and muscle impairment confers a clearly greater frailty burden overall [29].

Several implications arise from this pattern. First, DXA alone may miss early treatment effects when muscle function improves before measurable changes in appendicular lean mass appear. Second, CT‐derived indices may detect clinically relevant remodelling that links early biologic change to later performance gains. Third, denosumab may be particularly relevant in patients with an osteosarcopenic phenotype, in whom both skeletal preservation and muscle‐related effects have direct clinical importance. Importantly, the between‐group differences in the primary functional outcomes remained consistent after propensity score weighting, suggesting that the observed associations were not fully explained by the measured factors influencing treatment selection.

Several limitations should be considered. The mediation analyses were exploratory and should be interpreted as hypothesis‐generating rather than causal evidence for a biomarker‐response pathway. The observed OPG/sRANKL response patterns are therefore best interpreted as statistically compatible with partial mediation, not as evidence that this pathway directly explained the later functional or structural benefits. Although major measured factors influencing treatment selection and recorded physical activity, resistance exercise and rehabilitation exposures were accounted for in sensitivity analyses, residual confounding from other unmeasured clinical or lifestyle factors and comorbidities cannot be excluded. In addition, differences across studies in comparator selection, follow‐up duration, diagnostic definitions and study setting likely contribute to variation in the reported muscle effects across cohorts.

In summary, compared with zoledronic acid, denosumab was associated with stronger early activation of the OPG/sRANKL axis, earlier improvement in muscle function, coordinated CT‐defined remodelling, later gains in DXA‐defined muscle mass and a lower study‐defined sarcopenia burden under the AWGS 2025‐informed framework. Viewed alongside current mechanistic and clinical evidence, these findings support further prospective evaluation of RANKL inhibition as a treatment strategy for osteosarcopenic phenotypes.

## Funding

This study was supported by the National Natural Science Foundation of China (82502296).

## Ethics Statement

This study was approved by the Health Sciences Institutional Review Board of Huashan Hospital, Fudan University (HIRB; approval no. 2019‐410) and was conducted in accordance with the 1964 Declaration of Helsinki and its later amendments. All participants provided written informed consent before enrollment.

## Conflicts of Interest

The authors declare no conflicts of interest.

## Supporting information


**Table S1:** Additional baseline characteristics related to treatment selection.
**Table S2:** Association between changes in bone mineral density and grip strength.
**Table S3:** Age‐stratified analysis of 36‐month changes in primary functional outcomes.
**Table S4:** Adjusted linear mixed‐effects models for major continuous outcomes.
**Table S5:** Longitudinal physical activity, resistance exercise, and physical therapy/rehabilitation.
**Table S6:** Lifestyle‐adjusted linear mixed‐effects models for primary functional outcomes.
**Table S7:** Covariate balance before and after propensity score weighting.
**Table S8:** Propensity score‐weighted sensitivity analysis of primary functional outcomes.

## Data Availability

The data that support the findings of this study are available from the corresponding author upon reasonable request.
