# Supplementary material for: Denosumab Versus Zoledronic Acid for Muscle Strength and Physical Performance in Postmenopausal Osteoporosis: A 3‐Year Prospective Study
Source: J Cachexia Sarcopenia Muscle. 2026 Oct 21;17(5):e70406. doi: 10.1002/jcsm.70406 (PMC13685528; doi:10.1002/jcsm.70406)
Supplement: Supplementary file 1 [file JCSM-17-e70406-s001.docx]

**Table S1. Additional Baseline Characteristics Related to Treatment Selection**

| **Characteristic** | **Denosumab (n = 82)** | **Zoledronic acid (n = 104)** | **P value** |
| --- | --- | --- | --- |
| Estimated creatinine clearance, mL/min | 75.5 ± 18.0 | 77.0 ± 25.8 | 0.634 |
| Prior bisphosphonate intolerance or inadequate response | 22/82 (26.8%) | 15/104 (14.4%) | 0.042* |
| Anticipated treatment continuity | 72/82 (87.8%) | 86/104 (82.7%) | 0.411 |
| **Administration preference** |  |  | 0.001** |
| No strong preference | 48/82 (58.5%) | 63/104 (60.6%) |  |
| Prefers semiannual subcutaneous administration | 25/82 (30.5%) | 13/104 (12.5%) |  |
| Prefers annual intravenous administration | 9/82 (11.0%) | 28/104 (26.9%) |  |

Values are mean ± SD or n/N (%). The P value for administration preference represents the overall comparison across the three categories. Estimated creatinine clearance was derived from existing baseline clinical data; the other treatment-selection characteristics were recorded as baseline covariates for the sensitivity analysis. * P < 0.05; ** P < 0.01; *** P < 0.001.

**Table S2. Association Between Changes in Bone Mineral Density and Grip Strength**

| **Time** | **BMD measure** | **Analysis** | **N** | **r or adjusted β** | **95% CI** | **P value** |
| --- | --- | --- | --- | --- | --- | --- |
| 12 months | Lumbar spine BMD | Pooled | 167 | 0.347 | 0.206 to 0.474 | <0.001*** |
|  |  | Denosumab | 76 | 0.092 | −0.137 to 0.311 | 0.431 |
|  |  | Zoledronic acid | 91 | −0.053 | −0.257 to 0.154 | 0.615 |
|  |  | Adjusted pooled (treatment group + age) | 167 | 1.673 | −5.363 to 8.709 | 0.641 |
|  | Femoral neck BMD | Pooled | 167 | −0.011 | −0.162 to 0.141 | 0.890 |
|  |  | Denosumab | 76 | −0.064 | −0.286 to 0.163 | 0.580 |
|  |  | Zoledronic acid | 91 | 0.040 | −0.168 to 0.243 | 0.710 |
|  |  | Adjusted pooled (treatment group + age) | 167 | −0.842 | −11.772 to 10.089 | 0.880 |
| 24 months | Lumbar spine BMD | Pooled | 147 | 0.471 | 0.334 to 0.588 | <0.001*** |
|  |  | Denosumab | 67 | 0.041 | −0.201 to 0.279 | 0.740 |
|  |  | Zoledronic acid | 80 | −0.098 | −0.311 to 0.124 | 0.385 |
|  |  | Adjusted pooled (treatment group + age) | 147 | −2.128 | −10.728 to 6.472 | 0.628 |
|  | Femoral neck BMD | Pooled | 147 | −0.150 | −0.305 to 0.012 | 0.069 |
|  |  | Denosumab | 67 | 0.121 | −0.123 to 0.351 | 0.330 |
|  |  | Zoledronic acid | 80 | −0.043 | −0.260 to 0.179 | 0.708 |
|  |  | Adjusted pooled (treatment group + age) | 147 | 4.312 | −9.682 to 18.306 | 0.546 |
| 36 months | Lumbar spine BMD | Pooled | 129 | 0.417 | 0.263 to 0.550 | <0.001*** |
|  |  | Denosumab | 60 | 0.100 | −0.158 to 0.345 | 0.447 |
|  |  | Zoledronic acid | 69 | −0.008 | −0.244 to 0.230 | 0.951 |
|  |  | Adjusted pooled (treatment group + age) | 129 | 2.930 | −8.705 to 14.565 | 0.622 |
|  | Femoral neck BMD | Pooled | 129 | −0.261 | −0.415 to −0.092 | 0.003** |
|  |  | Denosumab | 60 | 0.012 | −0.243 to 0.265 | 0.929 |
|  |  | Zoledronic acid | 69 | −0.084 | −0.314 to 0.156 | 0.494 |
|  |  | Adjusted pooled (treatment group + age) | 129 | −2.409 | −15.538 to 10.720 | 0.719 |

Pooled and treatment-group-specific estimates are Pearson correlation coefficients (r) between change in BMD and change in grip strength at the corresponding follow-up visit. Adjusted pooled estimates are unstandardized regression coefficients (β) for BMD change from linear regression models additionally adjusted for treatment group and age; therefore, their numerical scale differs from that of r. BMD, bone mineral density; CI, confidence interval. * P < 0.05; ** P < 0.01; *** P < 0.001.

**Table S3. Age-Stratified Analysis of 36-Month Changes in Primary Functional Outcomes**

| **Outcome** | **Age stratum** | **Denosumab, n** | **36-month change, mean ± SD** | **Zoledronic acid, n** | **36-month change, mean ± SD** | **Between-group difference** | **P value** | **P for interaction** |
| --- | --- | --- | --- | --- | --- | --- | --- | --- |
| Grip strength, kg | 50–64 years | 39 | 2.55 ± 0.64 | 43 | 0.99 ± 0.44 | 1.56 | <0.001*** | 0.705 |
|  | ≥65 years | 21 | 2.46 ± 0.61 | 26 | 0.98 ± 0.47 | 1.48 | <0.001*** |  |
| Gait speed, m/s | 50–64 years | 39 | 0.071 ± 0.017 | 43 | 0.028 ± 0.012 | 0.043 | <0.001*** | 0.770 |
|  | ≥65 years | 21 | 0.070 ± 0.016 | 26 | 0.025 ± 0.012 | 0.044 | <0.001*** |  |
| 5-times chair stand, s | 50–64 years | 39 | −1.51 ± 0.30 | 43 | −0.68 ± 0.25 | −0.83 | <0.001*** | 0.341 |
|  | ≥65 years | 21 | −1.55 ± 0.27 | 26 | −0.63 ± 0.23 | −0.92 | <0.001*** |  |

Values are mean ± SD for change from baseline to 36 months. Between-group differences are denosumab minus zoledronic acid. Negative differences in 5-times chair stand time favor denosumab. P for interaction tests the treatment-by-age-stratum interaction for each functional outcome. * P < 0.05; ** P < 0.01; *** P < 0.001.

**Table S4. Adjusted Linear Mixed-Effects Models for Major Continuous Outcomes**

| **Outcome** | **12 months, adjusted difference (95% CI)** | **P (12 mo)** | **24 months, adjusted difference (95% CI)** | **P (24 mo)** | **36 months, adjusted difference (95% CI)** | **P (36 mo)** | **Adjusted P for group × time** |
| --- | --- | --- | --- | --- | --- | --- | --- |
| Lumbar spine BMD, g/cm² | 0.009 (0.007, 0.012) | <0.001*** | 0.013 (0.010, 0.015) | <0.001*** | 0.008 (0.005, 0.011) | <0.001*** | 0.036* |
| Femoral neck BMD, g/cm² | -0.000 (-0.002, 0.002) | 0.891 | -0.003 (-0.005, -0.001) | 0.003** | -0.004 (-0.006, -0.002) | <0.001*** | 0.012** |
| Whole-body DXA ASMI, kg/m² | 0.013 (-0.004, 0.030) | 0.144 | 0.083 (0.065, 0.102) | <0.001*** | 0.101 (0.082, 0.121) | <0.001*** | <0.001*** |
| Grip strength, kg | 0.810 (0.661, 0.959) | <0.001*** | 1.263 (1.105, 1.422) | <0.001*** | 1.560 (1.394, 1.727) | <0.001*** | <0.001*** |
| Gait speed, m/s | 0.024 (0.020, 0.028) | <0.001*** | 0.039 (0.034, 0.043) | <0.001*** | 0.043 (0.039, 0.048) | <0.001*** | <0.001*** |
| 5-times chair stand, s | -0.494 (-0.574, -0.414) | <0.001*** | -0.803 (-0.888, -0.718) | <0.001*** | -0.848 (-0.938, -0.758) | <0.001*** | <0.001*** |
| OPG/sRANKL ratio | 1.984 (1.536, 2.433) | <0.001*** | 3.646 (3.181, 4.111) | <0.001*** | 4.511 (4.032, 4.990) | <0.001*** | <0.001*** |
| T12-L4 SMVI | 0.721 (0.539, 0.902) | <0.001*** | 1.442 (1.249, 1.635) | <0.001*** | 1.872 (1.665, 2.078) | <0.001*** | <0.001*** |
| T12-L4 SFI | 0.065 (-0.379, 0.510) | 0.774 | 0.245 (-0.231, 0.720) | 0.314 | 0.276 (-0.237, 0.789) | 0.292 | 0.777 |
| T12-L4 VFI | -0.287 (-0.673, 0.100) | 0.146 | -0.763 (-1.178, -0.349) | <0.001*** | -0.788 (-1.235, -0.341) | <0.001*** | 0.139 |
| T12-L4 IMFI | -0.062 (-0.090, -0.035) | <0.001*** | -0.129 (-0.158, -0.100) | <0.001*** | -0.178 (-0.209, -0.147) | <0.001*** | <0.001*** |
| T12-L4 SMD | 0.972 (0.742, 1.202) | <0.001*** | 1.588 (1.344, 1.831) | <0.001*** | 2.083 (1.825, 2.341) | <0.001*** | <0.001*** |

Values are adjusted between-group differences (Denosumab minus zoledronic acid) in change from baseline at each follow-up visit, estimated from linear mixed-effects models with a subject-specific random intercept. Sensitivity models additionally included age, baseline vitamin D supplementation, and baseline variables showing between-group imbalance (baseline BDNF, P3NP, TNF-α, and T12-L4 VFI); the VFI model did not include baseline VFI as a covariate. Reductions in 5-times chair stand time, VFI, and IMFI favor denosumab when the adjusted between-group difference is negative. All models used available-case longitudinal data for the relevant outcome. * P < 0.05; ** P < 0.01; *** P < 0.001.

**Table S5. Longitudinal Physical Activity, Resistance Exercise, and Physical Therapy/Rehabilitation**

| **Variable** | **Time** | **Denosumab** | **Zoledronic acid** | **P value** |
| --- | --- | --- | --- | --- |
| Regular physical activity | Baseline | 48/82 (58.5%) | 58/104 (55.8%) | 0.766 |
|  | 12 months | 45/76 (59.2%) | 50/91 (54.9%) | 0.639 |
|  | 24 months | 38/67 (56.7%) | 50/80 (62.5%) | 0.503 |
|  | 36 months | 32/60 (53.3%) | 39/69 (56.5%) | 0.727 |
| Resistance exercise | Baseline | 18/82 (22.0%) | 21/104 (20.2%) | 0.856 |
|  | 12 months | 18/76 (23.7%) | 23/91 (25.3%) | 0.858 |
|  | 24 months | 11/67 (16.4%) | 17/80 (21.2%) | 0.530 |
|  | 36 months | 9/60 (15.0%) | 7/69 (10.1%) | 0.434 |
| Physical therapy/rehabilitation | Baseline | 11/82 (13.4%) | 9/104 (8.7%) | 0.345 |
|  | 12 months | 4/76 (5.3%) | 7/91 (7.7%) | 0.756 |
|  | 24 months | 4/67 (6.0%) | 6/80 (7.5%) | 0.755 |
|  | 36 months | 4/60 (6.7%) | 8/69 (11.6%) | 0.379 |

Values are n/N (%). Between-group comparisons used Fisher exact tests. Follow-up denominators reflect participants with available data at the corresponding visit. * P < 0.05; ** P < 0.01; *** P < 0.001.

**Table S6. Lifestyle-Adjusted Linear Mixed-Effects Models for Primary Functional Outcomes**

| **Outcome** | **Metric** | **Unadjusted estimate** | **Unadjusted P** | **Lifestyle-adjusted estimate** | **Adjusted P** |
| --- | --- | --- | --- | --- | --- |
| Grip strength, kg | 12-mo treatment difference | 0.779 | <0.001*** | 0.769 | <0.001*** |
|  | 24-mo treatment difference | 1.218 | <0.001*** | 1.218 | <0.001*** |
|  | 36-mo treatment difference | 1.510 | <0.001*** | 1.502 | <0.001*** |
|  | Global group × time |  | <0.001*** |  | <0.001*** |
| Gait speed, m/s | 12-mo treatment difference | 0.0237 | <0.001*** | 0.0236 | <0.001*** |
|  | 24-mo treatment difference | 0.0381 | <0.001*** | 0.0382 | <0.001*** |
|  | 36-mo treatment difference | 0.0426 | <0.001*** | 0.0427 | <0.001*** |
|  | Global group × time |  | <0.001*** |  | <0.001*** |
| 5-times chair stand, s | 12-mo treatment difference | −0.502 | <0.001*** | −0.502 | <0.001*** |
|  | 24-mo treatment difference | −0.796 | <0.001*** | −0.802 | <0.001*** |
|  | 36-mo treatment difference | −0.840 | <0.001*** | −0.848 | <0.001*** |
|  | Global group × time |  | <0.001*** |  | <0.001*** |

Regular physical activity, resistance exercise, and physical therapy/rehabilitation were included as time-varying covariates in the lifestyle-adjusted linear mixed-effects models. Treatment differences are denosumab minus zoledronic acid for change from baseline. Negative differences in 5-times chair stand time favor denosumab. * P < 0.05; ** P < 0.01; *** P < 0.001.

**Table S7. Covariate Balance Before and After Propensity Score Weighting**

| **Covariate** | **SMD before weighting** | **SMD after weighting** |
| --- | --- | --- |
| Age | -0.257 | -0.110 |
| Prior fragility fracture | 0.023 | 0.065 |
| Lumbar spine BMD | 0.141 | 0.103 |
| Femoral neck BMD | -0.089 | -0.008 |
| Estimated creatinine clearance | -0.069 | 0.025 |
| Baseline vitamin D supplementation | 0.333 | -0.060 |
| Prior bisphosphonate intolerance or inadequate response | 0.309 | 0.045 |
| Anticipated treatment continuity | 0.144 | -0.026 |
| Prefers semiannual subcutaneous administration | 0.446 | 0.065 |
| Prefers annual intravenous administration | -0.413 | 0.102 |

The propensity score for receipt of denosumab was estimated using age, prior fragility fracture, baseline lumbar spine and femoral neck BMD, estimated creatinine clearance, baseline vitamin D supplementation, prior bisphosphonate intolerance or inadequate response, anticipated treatment continuity, and administration preference. Administration preference was represented by indicator variables with no strong preference as the reference category. A stabilized inverse probability of treatment weighting (IPTW) approach was used. The propensity score model c-statistic was 0.736; stabilized weights ranged from 0.464 to 5.895, and the effective sample size was 140.2. The maximum absolute SMD decreased from 0.446 before weighting to 0.110 after weighting. BMD, bone mineral density; SMD, standardized mean difference.

**Table S8. Propensity Score-Weighted Sensitivity Analysis of Primary Functional Outcomes**

| **Outcome** | **Follow-up** | **N** | **Unweighted difference (95% CI)** | **P value** | **IPTW-weighted difference (95% CI)** | **P value** |
| --- | --- | --- | --- | --- | --- | --- |
| Grip strength, kg | 12 months | 167 | 0.790 (0.655 to 0.924) | <0.001*** | 0.768 (0.633 to 0.903) | <0.001*** |
|  | 24 months | 147 | 1.238 (1.065 to 1.411) | <0.001*** | 1.263 (1.069 to 1.457) | <0.001*** |
|  | 36 months | 129 | 1.530 (1.338 to 1.723) | <0.001*** | 1.607 (1.386 to 1.828) | <0.001*** |
| Gait speed, m/s | 12 months | 167 | 0.024 (0.020 to 0.027) | <0.001*** | 0.024 (0.019 to 0.028) | <0.001*** |
|  | 24 months | 147 | 0.039 (0.034 to 0.043) | <0.001*** | 0.035 (0.029 to 0.042) | <0.001*** |
|  | 36 months | 129 | 0.043 (0.038 to 0.049) | <0.001*** | 0.045 (0.040 to 0.051) | <0.001*** |
| 5-times chair stand, s | 12 months | 167 | -0.504 (-0.575 to -0.433) | <0.001*** | -0.493 (-0.569 to -0.417) | <0.001*** |
|  | 24 months | 147 | -0.819 (-0.916 to -0.722) | <0.001*** | -0.798 (-0.895 to -0.701) | <0.001*** |
|  | 36 months | 129 | -0.860 (-0.953 to -0.767) | <0.001*** | -0.863 (-0.953 to -0.772) | <0.001*** |

Values are between-group differences (denosumab minus zoledronic acid) in change from baseline at each follow-up visit, with 95% confidence intervals. The IPTW analysis used stabilized weights derived from the propensity score model described in Table S7. Negative differences in 5-times chair stand time favor denosumab. CI, confidence interval; IPTW, inverse probability of treatment weighting. * P < 0.05; ** P < 0.01; *** P < 0.001.
